# Exploring the biodiversity of *Bifidobacterium asteroides* among honey bee microbiomes

**DOI:** 10.1111/1462-2920.16223

**Published:** 2022-10-03

**Authors:** Gabriele Andrea Lugli, Federico Fontana, Chiara Tarracchini, Leonardo Mancabelli, Christian Milani, Francesca Turroni, Marco Ventura

**Affiliations:** ^1^ Laboratory of Probiogenomics, Department of Chemistry, Life Sciences, and Environmental Sustainability University of Parma Parma Italy; ^2^ Department of Medicine and Surgery University of Parma Parma Italy; ^3^ Microbiome Research Hub University of Parma Parma Italy

## Abstract

*Bifidobacterium asteroides* is considered the ancestor of the genus *Bifidobacterium*, which has evolved in close touch with the hindgut of social insects. However, recent studies revealed high intraspecies biodiversity within this taxon, uncovering the putative existence of multiple bifidobacterial species, thus, suggesting its reclassification. Here, a genomic investigation of 98 *B. asteroides*‐related genomes retrieved from public repositories and reconstructed from metagenomes of the hindgut of *Apis mellifera* and *Apis cerana* was performed to shed light on the genetic variability of this taxon. Phylogenetic and genomic analyses revealed the existence of eight clusters, of which five have been recently characterized with a representative type strain of the genus and three were represented by putative novel bifidobacterial species inhabiting the honeybee gut. Then, the dissection of 366 shotgun metagenomes of honeybee guts revealed a pattern of seven *B. asteroides*‐related taxa within *A. mellifera* that co‐exist with the host, while *A. cerana* microbiome was characterized by the predominance of one of the novel species erroneously classified as *B. asteroides*. A further glycobiome analysis unveiled a conserved repertoire of glycosyl hydrolases (GHs) reflecting degradative abilities towards a broad range of simple carbohydrates together with genes encoding specific GHs of each *B. asteroides*‐related taxa.

## INTRODUCTION

Members of the genus *Bifidobacterium* are Gram‐positive bacteria subjected to growing interest due to their health‐promoting properties exerted on human health (Hidalgo‐Cantabrana et al., 2017; Milani et al., 2017; Ventura et al., 2014). They are mainly distributed in the gut of mammals (Turroni et al., 2018), but they can also be identified in the hindgut of social insects, oral cavities, sewage, blood and foods (Bunesova et al., 2014). The advent of the genomic era allowed to easily decode the genome sequences of bifidobacteria, providing a tool to shed light on genetics and phylogenetics of these microorganisms (Lugli et al., 2014; Milani et al., 2014). Furthermore, a combination of genome sequencing and bacterial isolation attempts expanded our knowledge regarding the genus *Bifidobacterium* through the identification and characterization of more than 100 different bifidobacterial species (Lugli, Alessandri, et al., 2021; Lugli, Calvete‐Torre, et al., 2021; Lugli, Duranti, et al., 2019; Lugli, Milani, et al., 2019). Remarkably, in the last 10 years, more bifidobacterial species have been described than in the previous century, starting from the identification of the first *Bifidobacterium bifidum* in 1899 by Henri Tissier from the intestinal microbiota of a breastfed infant (Tissier, 1900).

So far, several members of the genus *Bifidobacterium* have been characterized from the guts of different social insects, highlighting a rich bifidobacterial‐related environment second only to the gut of mammals (Bunesova et al., 2014). Four species were identified from the guts of bumble bees (*Bombus lucorum*, *Bombus lapidarius*, *Bombus terrestris*, and *Bombus hypnorum*), named after *Bifidobacterium actinocoloniiforme*, *Bifidobacterium bohemicum*, *Bifidobacterium bombi* and *Bifidobacterium commune* (Killer et al., 2009, 2011; Praet et al., 2015). Furthermore, *Bifidobacterium aemilianum* and *Bifidobacterium xylocopae* were isolated from the guts of *Xylocopa violacea*, the violet carpenter bee (Alberoni et al., 2019). In addition, among two of the eight species of honey bees, that is, *Apis mellifera* (the western honey bee) and *Apis cerana* (the eastern honey bee) (Danforth, 2007), three bifidobacterial species were characterized, that is, *Bifidobacterium asteroides*, *Bifidobacterium coryneforme* and *Bifidobacterium indicum* (Milani et al., 2014).

Among the species inhabiting the gut of social insects, *B. asteroides* was subjected to genomic investigation predicting its capability for a putative respiratory metabolism (Bottacini et al., 2012). This notion was also investigated from an evolutionary perspective highlighting that respiration is a common metabolic feature of this ancient bifidobacterial species, which has been lost in mammal‐derived *Bifidobacterium* species (Bottacini et al., 2012). Furthermore, the glycan‐degrading abilities of *B. asteroides* have been investigated in respect to those of the other members of the genus, highlighting a more extended adaptation history to their hosts that resulted in a different repertoire of glycosyl hydrolases (GHs) characterized by GH43 and GH3 enzymes (Milani et al., 2015).

More recently, four novel *Bifidobacterium* species were isolated from honey bees, named after *Bifidobacterium apousia*, *Bifidobacterium choladohabitans*, *Bifidobacterium mizhiense* and *Bifidobacterium polysaccharolyticum*, whose genomic investigation revealed a close genetic relatedness towards *B. asteroides* type strain DSM 20089 (Chen et al., 2021; Li et al., 2022). Furthermore, based on genomic investigation aiming at exploring the intraspecies genetic diversity of members of the genus *Bifidobacterium*, *B. asteroides* was fragmented into four putative distinct bifidobacterial species (Lugli et al., 2018), resulting in the most complex *Bifidobacterium* taxon identified to date. Lately, different *B. asteroides* clusters have been confirmed by strain‐level investigation of the honey bee gut microbiota (Wu et al., 2021), requiring further investigation to disentangle the high biodiversity of this widespread taxon in the gut of *A. mellifera*.

In this study, we report an exhaustive genomic and metagenomic dissection of *B. asteroides* genomes retrieved from public repositories as well as reconstructed from the metagenomes of their hosts, that is, *A. mellifera* and *A. cerana*, allowing to redesign the *B. asteroides* phylogeny. Furthermore, to explore the distribution of *B. asteroides*‐related taxa among their shared hosts, 366 honey bee metagenomes (Regan et al., 2018; Wu et al., 2021, 2022) were investigated to uncover their ecological and evolutionary dynamics.

## EXPERIMENTAL PROCEDURES

### *Bifidobacterium* genome sequence selection and quality control

Complete and partial genomes of 17 *B. asteroides* strains, and 170 unclassified bifidobacterial strains, were retrieved from the NCBI database (Tables 1 and S1). Then, the genome sequence of *B. asteroides* DSM 20089, together with those of bifidobacteria isolated from social insects (Table 1), was used to discard those strains showing an average nucleotide identity (ANI) lower than 94% employing the software fastANI (Jain et al., 2018). Amino acid sequences of coding genes were predicted using Prodigal (Hyatt et al., 2010) and then used for further genomic analyses, while the quality of genomes was estimated for completeness and contamination using CheckM (Parks et al., 2015) (Table S3).

**TABLE 1:** *Bifidobacterium asteroides*‐related genomes.

NCBI code	Strain name	Predicted species	Cluster	Host
GCA_003202695.1	*Bifidobacterium asteroides* ESL0200	*Bifidobacterium* sp. nov.	CL1	*Apis mellifera*
GCA_019469425.1	*Bifidobacterium asteroides* ESL0447	*Bifidobacterium* sp. nov.	CL2	*Apis cerana*
GCA_016102005.1	*Bifidobacterium choladohabitans* JCM 34586	*Bifidobacterium choladohabitans*	CL3	*Apis mellifera*
GCA_000499285.1	*Bifidobacterium* sp. 7101	*Bifidobacterium choladohabitans*	CL3	*Apis mellifera*
GCA_000967265.1	*Bifidobacterium asteroides* Bin7	*Bifidobacterium choladohabitans*	CL3	*Apis mellifera*
GCA_009683175.1	*Bifidobacterium asteroides* VRA_9sq_n	*Bifidobacterium choladohabitans*	CL3	*Apis mellifera*
GCA_016100645.1	*Bifidobacterium* sp. M0353	*Bifidobacterium choladohabitans*	CL3	*Apis mellifera*
GCA_016100655.1	*Bifidobacterium* sp. W8107	*Bifidobacterium choladohabitans*	CL3	*Apis mellifera*
GCA_016100735.1	*Bifidobacterium* sp. W8104	*Bifidobacterium choladohabitans*	CL3	*Apis mellifera*
GCA_016100765.1	*Bifidobacterium* sp. W8105	*Bifidobacterium choladohabitans*	CL3	*Apis mellifera*
GCA_016100775.1	*Bifidobacterium* sp. W8103	*Bifidobacterium choladohabitans*	CL3	*Apis mellifera*
GCA_020884755.1	*Bifidobacterium mizhiense* JCM 34710	*Bifidobacterium mizhiense*	CL4	*Apis mellifera*
GCA_000499185.1	*Bifidobacterium* sp. A11	*Bifidobacterium mizhiense*	CL4	*Apis mellifera*
GCA_003688305.1	*Bifidobacterium* sp. wkB344	*Bifidobacterium mizhiense*	CL4	*Apis mellifera*
GCA_007559275.1	*Bifidobacterium apousia* JCM 34587	*Bifidobacterium apousia*	CL5	*Apis mellifera*
GCA_000967185.1	*Bifidobacterium asteroides* Bin2	*Bifidobacterium apousia*	CL5	*Apis mellifera*
GCA_002846895.1	*Bifidobacterium asteroides* 1460B	*Bifidobacterium apousia*	CL5	*Apis mellifera*
GCA_016100785.1	*Bifidobacterium* sp. W8120	*Bifidobacterium apousia*	CL5	*Apis mellifera*
GCA_016101425.1	*Bifidobacterium* sp. W8112	*Bifidobacterium apousia*	CL5	*Apis mellifera*
GCA_002715865.1	*Bifidobacterium asteroides* DSM 20089	*Bifidobacterium asteroides*	CL6	*Apis mellifera*
GCA_000304215.1	*Bifidobacterium asteroides* PRL2011	*Bifidobacterium asteroides*	CL6	*Apis mellifera*
GCA_003202855.1	*Bifidobacterium asteroides* ESL0170	*Bifidobacterium asteroides*	CL6	*Apis mellifera*
GCA_016101165.1	*Bifidobacterium* sp. W8114	*Bifidobacterium asteroides*	CL6	*Apis mellifera*
GCA_016101375.1	*Bifidobacterium* sp. W8110	*Bifidobacterium asteroides*	CL6	*Apis mellifera*
GCA_016102155.1	*Bifidobacterium asteroides* W8130	*Bifidobacterium asteroides*	CL6	*Apis mellifera*
GCA_016102205.1	*Bifidobacterium asteroides* W8118	*Bifidobacterium asteroides*	CL6	*Apis mellifera*
GCA_016102215.1	*Bifidobacterium asteroides* W8109	*Bifidobacterium asteroides*	CL6	*Apis mellifera*
GCA_003202755.1	*Bifidobacterium asteroides* ESL0199	*Bifidobacterium* sp. nov.	CL7	*Apis mellifera*
GCA_016101585.1	*Bifidobacterium polysaccharolyticum* JCM 34588	*Bifidobacterium polysaccharolyticum*	CL8	*Apis mellifera*
GCA_000970835.1	*Bifidobacterium asteroides* Hma3	*Bifidobacterium polysaccharolyticum*	CL8	*Apis mellifera*
GCA_003202715.1	*Bifidobacterium asteroides* ESL0198	*Bifidobacterium polysaccharolyticum*	CL8	*Apis mellifera*
GCA_003688325.1	*Bifidobacterium* sp. wkB338	*Bifidobacterium polysaccharolyticum*	CL8	*Apis mellifera*
GCA_016100595.1	*Bifidobacterium* sp. M0399	*Bifidobacterium polysaccharolyticum*	CL8	*Apis mellifera*
GCA_016101415.1	*Bifidobacterium* sp. M0404	*Bifidobacterium polysaccharolyticum*	CL8	*Apis mellifera*
GCA_017349335.1	*Bifidobacterium asteroides* wkB204	*Bifidobacterium polysaccharolyticum*	CL8	*Apis mellifera*
GCA_016100445.1	*Bifidobacterium* sp. M0307	*Bifidobacterium indicum/coryneforme*	‐	*Apis mellifera*
GCA_016102235.1	*Bifidobacterium* sp. W8108	*Bifidobacterium indicum/coryneforme*	‐	*Apis mellifera*
GCA_022484665.1	*Bifidobacterium* sp. UW_MP_BIF13_2	*Bifidobacterium* sp. nov.	‐	biological waste material
GCA_022486445.1	*Bifidobacterium* sp. UW_MP_BIF13_1	*Bifidobacterium* sp. nov.	‐	biological waste material
GCA_022647885.1	*Bifidobacterium* sp. UW_MP_BIF3_1	*Bifidobacterium* sp. nov.	‐	biological waste material
GCA_001263395.1	*Bifidobacterium actinocoloniiforme* DSM 22766	‐	‐	*Bombus*
GCA_003315635.1	*Bifidobacterium aemilianum* DSM 104956	‐	‐	*Xylocopa violacea*
GCA_000741525.1	*Bifidobacterium bohemicum* DSM 22767	‐	‐	*Bombus*
GCA_000737845.1	*Bifidobacterium bombi* DSM 19703	‐	‐	*Bombus*
GCA_900094885.1	*Bifidobacterium commune* R‐52791	‐	‐	*Bombus*
GCA_000737865.1	*Bifidobacterium coryneforme* LMG 18911	‐	‐	*Apis mellifera*
GCA_000706765.1	*Bifidobacterium indicum* LMG 11587	‐	‐	*Apis cerana*
GCA_003315615.1	*Bifidobacterium xylocopae* DSM 104955	‐	‐	*Xylocopa violacea*

### Phylogenomic comparison of *B. asteroides*‐related taxa

Collected genome sequences were subjected to a pangenome calculation using PGAP (Pan‐Genomes Analysis Pipeline) (Zhao et al., 2012). The proteome of all assessed genomes was organized into functional gene clusters using the GF (gene family) method, which involves a comparison of each protein to all other proteins using BLAST analysis, followed by clustering into clusters of orthologous genes (cut‐off *E*‐value of 1 × 10^−5^ and 50% identity across at least 80% of both protein sequences), using MCL (a graph‐theory‐based Markov cluster algorithm) (Enright et al., 2002). Protein families shared between all genomes, named core genes, were defined by selecting the families that contained at least one single protein member for each genome. The concatenated core genome sequences were aligned using MAFFT (multiple alignment using fast Fourier Transform) (Katoh et al., 2018), and the corresponding phylogenomic tree was constructed using the neighbour‐joining method in ClustalW version 2.1 (Li, 2003). The core genome tree was built using FigTree (http://tree.bio.ed.ac.uk/software/figtree/). For each genome pair, the ANI value was calculated using the software fastANI (Jain et al., 2018). Then, hierarchical clustering analysis (HCA) based on a matrix of ANI values between *Bifidobacterium* strains was constructed using OriginPro graphing and analysis 2021 (https://www.originlab.com/2021).

### Metagenome dataset selection

In this project, 366 publicly available datasets retrieved after 10 BioProject (listed in NCBI) from various geographical locations were obtained through honey bee hindgut sequencing literature (Table S3). In detail, we selected shotgun microbial profiling datasets, including microbiomes belonging to *A. mellifera* and *A. cerana* (Table S3).

### Taxonomic classification of the honey bee microbiome reads and whole metagenome assembly

To analyse high‐quality sequenced data only, each dataset was subjected to a filtering step removing low‐quality reads (minimum mean quality score 20, window size 5, quality threshold 25 and minimum length 100) using the fastq‐mcf script (https://github.com/ExpressionAnalysis/ea-utils/blob/wiki/FastqMcf.md). Filtered reads were then collected and taxonomically classified through the METAnnotatorX2 pipeline (Milani et al., 2021), using the up‐to‐date RefSeq (genome) database retrieved from the NCBI (https://www.ncbi.nlm.nih.gov/refseq/). Filtered reads were then subjected to whole metagenome assembly using Spades v3.15 (Bankevich et al., 2012) with default parameters and the metagenomic flag option (‐meta) together with k‐mer sizes of 21, 33, 55 and 77. As mentioned above, for the short reads, reconstructed contig sequences were taxonomically classified based on their sequence identity using megablast against the same RefSeq database (Chen et al., 2015). ORFs of each assembled genome were then predicted with Prodigal (Hyatt et al., 2010) and annotated utilizing the MEGAnnotator pipeline (Lugli et al., 2016). In all, the METAnnotatorX2 pipeline was employed for various purposes, from read filtering to taxonomic classification of the assembled contigs (Milani et al., 2018, 2021).

### Metagenome tracing of *B. asteroides*‐related taxa

Complete and partial genome sequences retrieved from NCBI and reclassified from CL1 to CL8 were used to trace their presence among the 366 publicly available datasets collected in this study. Previous filtered reads of each microbiome were then collected and taxonomically reclassified using a homemade RefSeq database with *B. asteroides* genomes subdivided into the eight CLs. Then, the reconstructed genome sequences obtained from the 366 samples after WMS assembly were reclassified following the updated database. Next, reconstructed genomes of *B. asteroides*‐related taxa were selected based on statistics (completeness >80% and contamination <3%) retrieved using the CheckM software (Parks et al., 2015). Each reconstructed genome was then validated using the 94% ANI threshold employing the software fastANI (Jain et al., 2018) and used for further phylogenomic and genetic analyses.

### *Bifidobacterium asteroides* glycobiome

The proteome of each reconstructed bifidobacterial strain was screened for genes predicted to encode carbohydrate‐active enzymes based on sequence similarity to genes classified in the carbohydrate‐active enzyme (CAZy) database (Drula et al., 2022). Thus, each predicted proteome of a given bifidobacterial strain was screened for orthologues against the dbCAN2 meta server (Zhang et al., 2018) composed of 2,141,452 CDS using HMMER v3.3.2 (cut‐off *E*‐value of 1 × 10^−15^ and coverage >0.35).

### Statistical analysis

Bacterial abundance at the species level between microbiomes was validated by ANOVA analysis. Furthermore, PERMANOVA analysis was performed using 1000 permutations to estimate *p*‐values of differences among samples in PCoA analyses. The hierarchical clustering analysis (HCA) of samples was performed using OriginPro graphing and analysis 2021 (https://www.originlab.com/2021), employing the Bray–Curtis matrix and Pearson correlation as a distance metric and the sum square of distances and furthest neighbour for clustering methods.

## RESULTS AND DISCUSSION

### Evaluation of intraspecies genomic variability of *Bifidobacterium asteroides* species

The analysis of the currently available *Bifidobacterium* genome sequences deposited in NCBI provided an exhaustive overview of the genomic diversity for each taxon. Seven species displayed more than a hundred sequenced genomes, representing to date the most studied bifidobacterial species, that is, *B. longum* (*n* = 674), *B. breve* (*n* = 204), *B. bifidum* (*n* = 178), *B. pseudocatenulatum* (*n* = 178), *B. animalis* (*n* = 123), *B. adolescentis* (*n* = 117), and *B. pseudolongum* (*n* = 107). Nonetheless, after the latter species, *B. catenulatum* (*n* = 57), *B. dentium* (*n* = 49) and finally *B. asteroides* (*n* = 17) showed the highest number of sequenced genomes, leaving behind additional 91 bifidobacterial species poorly characterized. This data highlighted an increasing interest of the scientific community about *B. asteroides*, probably due to the growing concerns regarding the health and behaviours of its common host, the honey bee.

**FIGURE 1:**
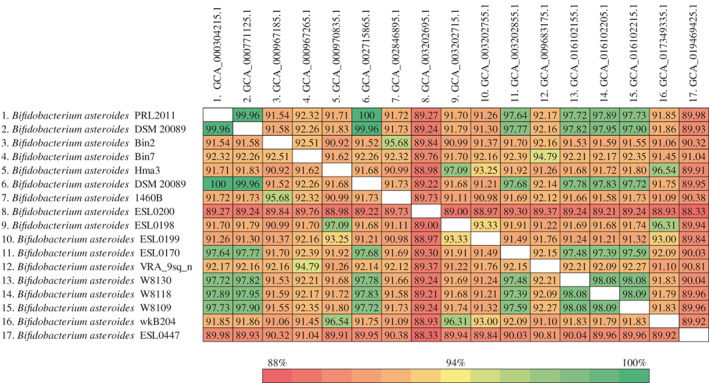
Average nucleotide identity (ANI) values between 17 *Bifidobacterium asteroides* strains retrieved from the NCBI database. Colour‐based scheme highlight those 10 strains with ANI values below the 94% threshold to be represented as members of the *B. asteroides* taxon.

The collection of 17 *B. asteroides* genome sequences from the NCBI database allowed us to investigate the genome variability of this taxon. Each genome was subjected to ANI evaluation against the other *B. asteroides* genome sequences. Such analyses revealed ANI values lower than 90% in respect to the *B. asteroides* type strain, that is, *B. asteroides* ESL0447 (ANI value of 89.9%) and *B. asteroides* ESL0200 (ANI value of 89.2%), which cast doubt on the correct taxonomic classification of these strains (Figure 1). In this regard, it should be noted that two strains displaying an ANI value of <95% are considered to belong to two distinct species (Konstantinidis et al., 2006; Richter & Rosselló‐Móra, 2009). Moreover, recent phylogenomics studies regarding the *Bifidobacterium* genus have demonstrated that 94% is an excellent ANI threshold to define bifidobacterial species boundaries (Lugli et al., 2017, 2018). Thus, 10 out of 17 strains were below this accepted threshold, showing that more than half of the submitted genomes may not represent members of the *B. asteroides* taxon (Figure 1).

The genome sequences of additional 170 bifidobacterial unclassified strains were collected from the NCBI repository to ensure an exhaustive screening of the genomic diversity of the *B. asteroides* taxon (Table S1). Thus, an ANI evaluation including genome sequences of these unclassified bifidobacteria altogether with 17 *B. asteroides* strains (*n* = 187) was performed to gather additional *B. asteroides*‐related genomes. In this context, a hierarchical clustering analysis employing the ANI values matrix of all 187 genome sequences allowed to identify 26 unclassified genomes related to the *B. asteroides* strains (Figure S1). Subsequently, sequence quality of the so‐gathered genomes was evaluated through the CheckM pipeline, discarding strains with completeness <85% and contamination >3% (Table S2). The process results in selecting 36 distinct strains related to *B. asteroides* with a suitable genome quality for further phylogenetic and metagenomic analyses (Table 1).

### Phylogenetic dissection of *B. asteroides‐*related strains revealed the existence of eight possible novel different taxa

**FIGURE 2:**
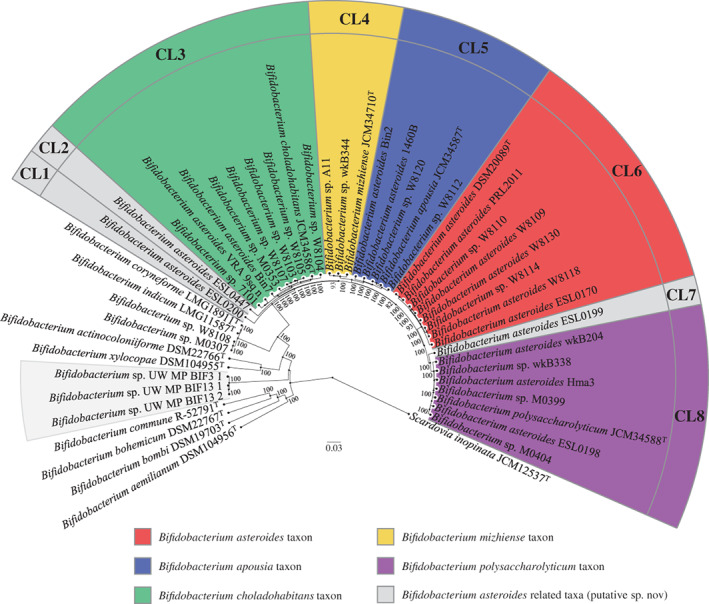
Phylogenomic tree of 48 *Bifidobacterium asteroides*‐related strains based on the concatenation of 318 core protein sequences. Different colours highlight the division into eight taxa (named from CL1 to CL8) between strains previously classified as *B. asteroides* or unclassified bifidobacterial species. The phylogenetic tree was constructed by the neighbour‐joining method, with the genome sequence of *Scardovia inopinata* JCM 12537 as an outgroup. Bootstrap percentages above 50 are shown at node points based on 1000 replicates of the phylogenetic tree.

Based on the recent scientific literature about the bifidobacterial ecology, *B. asteroides* was only one of those *Bifidobacterium* species identified and isolated from social insects, such as *A. mellifera*, *A. cerana*, *Bombus* and *Xylocopa violacea*. Thus, the type strains of the closed phylogenetic species to *B. asteroides*, that is, *Bifidobacterium actinocoloniiforme* DSM 22766, *B. aemilianum* DSM 104956, *B. apousia* JCM 34587, *B. bohemicum* DSM 22767, *B. bombi* DSM 19703, *B. choladohabitans* JCM 34586, *B. commune* R‐52791, *B. coryneforme* LMG 18911, *B. indicum* LMG 11587, *B. mizhiense* JCM 34710, *B. polysaccharolyticum* JCM 34588 and *B. xylocopae* DSM 104955, were added to the analysis. Gathered genomes were then employed to study the phylogeny of the 36 strains recognized as *B. asteroides*‐related strains (Table 1). The relationship between bifidobacterial species isolated from social insects was initially evaluated by performing gene prediction among the 48 genome sequences, avoiding inconsistencies due to different gene prediction methods. Then, a pangenome analysis of the collected strains was undertaken to determine putative orthologous genes between the 48 strains. The analysis identified 10,698 clusters of orthologous genes (COGs), representing the pangenome of strains isolated from social insects. Collected COGs allowed the identification of 370 genes shared between all analysed genomes. Furthermore, after exclusion of paralogs, the concatenation of 318 core protein sequences was used to build a *Bifidobacterium* phylogenomic tree using *Scardovia inopinata* JCM 12537 gene sequences as outgroup (Figure 2).

The phylogenetic reconstruction of bifidobacteria isolated from insects allowed to define eight clusters from CL1 to CL8 containing *Bifidobacterium* strains previously classified as *B. asteroides* highlighting divergent evolutionary routes (Figure 2). *B. asteroides* type strain fell in CL6 with only seven other bifidobacteria, representing those strains that belong to this taxon (Table 1). In contrast, all the other related strains were distributed among the other seven clusters except for *Bifidobacterium* sp. UW_MP_BIF3_1, *Bifidobacterium* sp. UW_MP_BIF13_1, and *Bifidobacterium* sp. UW_MP_BIF13_2, which cluster apart from any classified taxon (Figure 2). Interestingly, these latter strains were genomically related to *Bifidobacterium crudilactis* (ANI between 79.4 % and 80.1%), representing a putative novel species of the genus *Bifidobacterium*. However, since these strains do not belong to a *B. asteroides*‐related taxon, they were excluded from further analyses, as well as Bifidobacterium (*B)*. sp. W8108 and *B*. sp. M0307, which were found to be related to *B. coryneforme* LMG 18911 and *B. indicum* LMG 11587 (Figure 2).

Among the identified clusters, four of them were represented by *Bifidobacterium* taxa recently characterized, that is, *B. choladohabitans* JCM 34586 (CL3), *B. mizhiense* JCM 34710 (CL4), *B. apousia* JCM 34587 (CL5) and *B. polysaccharolyticum* JCM 34588 (CL8) (Chen et al., 2021; Li et al., 2022) (Figure 2). Thus, thanks to isolation attempts and genomic characterization of the isolates, the large part of the identified clusters were reclassified and a representative type strain defined. Nonetheless, three clusters (CL1, CL2 and CL7) still await an exhaustive characterization representing three putative novel bifidobacterial species inhabiting the honey bee's gut. Specifically, the latter clusters were composed of single strains, such as *B. asteroides* ESL0200 (CL1), *B. asteroides* ESL0447 (CL2) and *B. asteroides* ESL0199 (CL7). Thus, further investigation of the honey bee microbiome is necessary to better explore the *B. asteroides* biodiversity.

Altogether genomic and phylogenetic analyses highlighted that the intraspecies variability of the *B. asteroides* taxon is distributed among eight clusters. However, to date, only five clusters have been characterized with a representative type strain of the genus, while the remaining three clusters may represent putative novel bifidobacterial species inhabiting the gut of honey bees.

### Taxonomic classification of the honey bee microbiome

Since each representative strain of the eight *B. asteroides*‐related clusters was isolated from the gut of honey bees, we decided to explore its distribution by collecting *A. mellifera* and *A. cerana* metagenomics data from the short read archive (SRA). Thus, sequenced DNA of 314 *A. mellifera* and 52 *A. cerana* microbiomes was selected from 10 sequencing projects publicly available in the NCBI repository (Table S3). Collected data were subjected to microbial profiling based on short‐read taxonomic classification down to species level after filtering steps based on DNA sequence quality and host DNA removal (Table S4).

**FIGURE 3:**
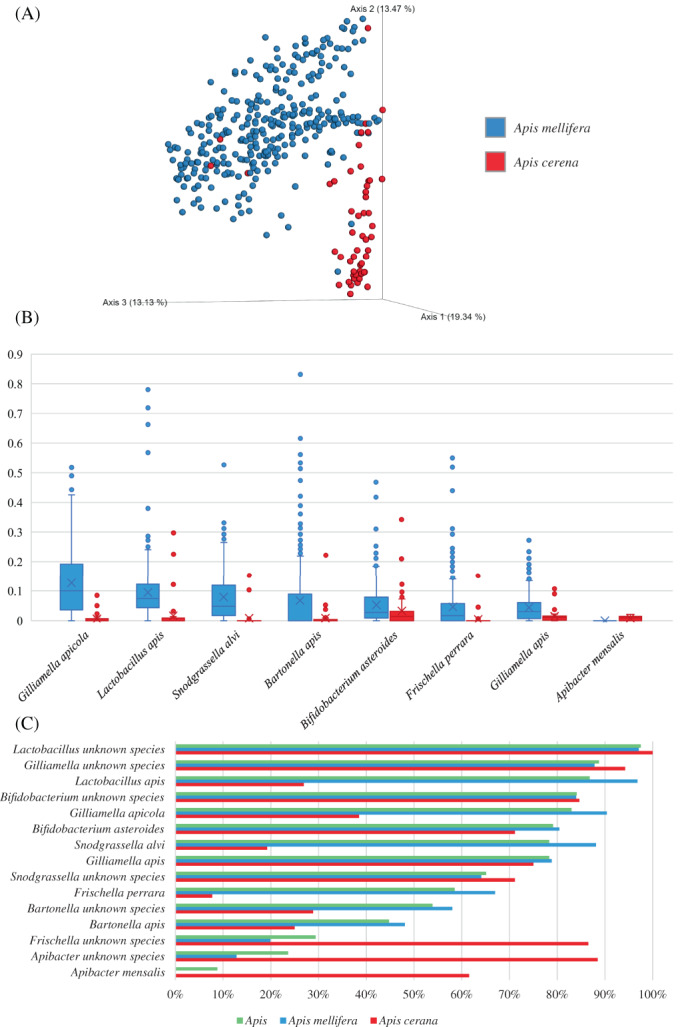
Taxonomic composition of 366 gut microbiomes of *Apis mellifera* and *Apis cerana*. (A) Shows a principal coordinate analysis (PCoA) based on microbial distribution using the Bray–Curtis index represented in different colours by means of *A. mellifera* (light blue) and *A. cerana* (red). (B) Displays a Whisker plot based on average abundances of main microbial constituents inhabiting the gut of *A. mellifera* (light blue) and *A. cerana* (red). The y‐axis shows the average abundance of each observation. Dots reflect the distribution of a data set, while the boxes represent 50% of the data set, distributed between the first and third quartiles. The median divides the boxes into the interquartile range, while the X represents the mean. The lines extending vertically outside the boxes show the outlier range. (C) Depicts the prevalent bacterial taxa identified among all processed samples (green), *A. mellifera* microbiomes (light blue), and *A. cerana* microbiomes (red).

In silico investigation uncovered that the most abundant species among honey bee microbiomes were represented by microorganisms typically associated with the host, such as *Bartonella apis*, *B. asteroides*, *Frischella perrara*, *Gilliamella apicola*, *Gilliamella apis*, *Lactobacillus apis* and *Snodgrassella alvi*. All the latter species were found with a prevalence higher than 45%, representing the core microbiota of honey bees (Figure 3). However, significant differences occurred by comparing the gut microbiota composition of *A. mellifera* in respect to that of *A. cerana* (PERMANOVA *p*‐value of <0.05), as depicted by a beta‐diversity investigation represented through principal coordinate analysis (PCoA) based on Bray–Curtis dissimilarity index (Figure 3). More in specific, with the exclusion of *B. asteroides* and *Gilliamella apicola*, the other five most prevalent taxa above reported were identified with a significant low prevalence in the gut of *A. cerana* (Figure 3). In contrast, *Apibacter mensalis* taxon was prevalent in 62% of the *A. cerana* samples and completely undetected in the microbiota of *A. mellifera*, highlighting a strict co‐evolutionary route of this microorganism and its host. In addition, the dissection of *A. mellifera* and *A. cerana* microbiomes revealed a number of putative unknown species that have not been classified so far with a prevalence >50% belonging to the genus *Lactobacillus*, *Bifidobacterium*, *Gilliamella*, *Snodgrassella*, and *Bartonella*, showing that the microbial biodiversity of the honey bee has not been fully explored (Figure 3).

Focusing on bifidobacteria, *B. asteroides* was the taxa with the highest average abundance of 5%, followed by *B. indicum* and *B. coryneforme* with average abundances of 0.26 and 0.25%, respectively. Notably, the near‐identical average abundance of the latter two taxa reflects the inability to discriminate between them since their ANI has been previously reported to be >98% (Lugli et al., 2014). Thus, based on this notion, it should be more accurate to state that *B. indicum*/*coryneforme* was identified with an average abundance of 0.5%. Furthermore, a portion of the honey bee microbiome was represented by unclassified species of the *Bifidobacterium* genus (average abundance of 6%), highlighting the gap in knowledge concerning bifidobacteria and honey bees (Figure 3). Interestingly, none of the bifidobacterial species isolated from other social insects was detected from the hindgut of *A. mellifera* and *A. cerana*, that is, *B. actinocoloniiforme*, *B. aemilianum*, *B. bohemicum*, *B. bombi*, *B. commune* and *B. xylocopae*. These findings highlighted a specialization of bifidobacteria for certain insects since no trace of the latter taxa were isolated from *Bombus* and *Xylocopa violacea* was identified in honey bees.

These findings confirmed that *B. asteroides* is the most common member of the genus *Bifidobacterium* harboured by *A. mellifera* and *A. cerana*. However, up‐to‐date bioinformatics pipelines cannot distinguish between the above predicted *B. asteroides‐*related clusters (CL1–CL8). For this reason, a further investigation was conducted to explore the actual distribution of all different putative species recognized as members of the *B. asteroides* taxon.

### Bifidobacterial distribution among *Apis* hindgut revealed a complex *Bifidobacterium*‐based ecosystem

An in‐house database was built encompassing representative strain of each *B. asteroides‐*related cluster to investigate their distribution among *A. mellifera* and *A. cerana*. In this context, the chromosomal sequences of the 35 reclassified *B. asteroides* were subdivided from CL1 to CL8, representing both described and putative novel species, that is, *B*. sp. nov. CL1, *B*. sp. nov. CL2, *B. choladohabitans* (CL3), *B. mizhiense* (CL4), *B. apousia* (CL5), *B. asteroides* (CL6), *B*. sp. nov. CL7 and *B. polysaccharolyticum* (CL8) (Table 1). Therefore, 366 honey bee microbiomes were re‐analysed allowing to taxonomically classify the previous DNA widely assigned to *B. asteroides* to the proper *B. asteroides* CL.

**FIGURE 4:**
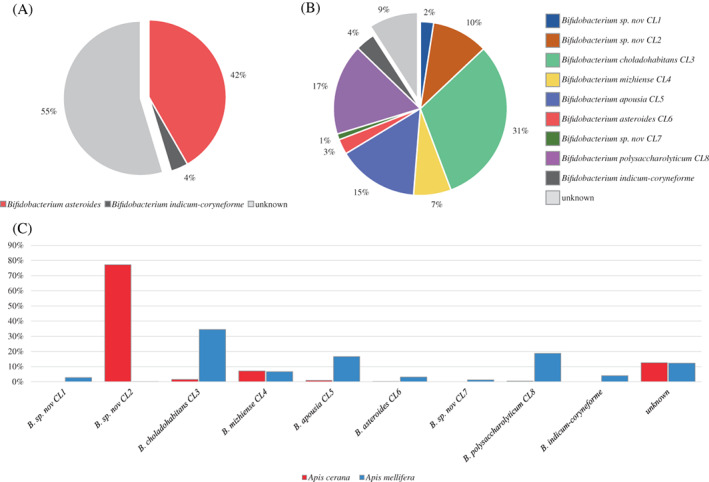
Distribution of *Bifidobacterium asteroides*‐related taxa among 366 gut microbiomes of *Apis*. *mellifera* and *Apis cerana*. (A) Exhibits the relative abundance of bifidobacteria at species level using a non‐up‐to‐date database for taxonomical classification of bifidobacteria, while panel B shows the distribution of *B. asteroides*‐related taxa using an updated database built using the reclassified genomes analysed in this project. (C) Displays the distribution of *B. asteroides*‐related taxa *A. mellifera* microbiomes (light blue) and *A. cerana* microbiomes (red).

Interestingly, collecting data taxonomically classified to members of the genus *Bifidobacterium*, the amount of predicted DNA belonging to unknown species of bifidobacteria was reduced from 55% to 9%, demonstrating that our in‐house database constituted by the eight *B. asteroides* CLs was able to disentangle 91% of bifidobacteria harboured by *A. mellifera* and *A. cerana* (Figure 4). Similarly, the amount of *B. asteroides* DNA was also reduced from 42% to 3% (*B. asteroides* [CL6]), revealing how limited was the contribution of the actual *B. asteroides* taxon to the microbiota variability of honey bees and uncovering a more complex bifidobacterial ecosystem. Instead, those bifidobacterial taxa highly distributed among honey bee microbiomes were represented by *B. choladohabitans* (CL3) (31%), *B. polysaccharolyticum* (CL8) (17%), *B. apousia* (CL5) (15%) and *B*. sp. nov. CL2 (10%) (Figure 4).

Comparing taxonomic data between *A. mellifera* and *A. cerana* microbiomes, a very diverse distribution of bifidobacteria emerged, showing *B. asteroides*‐related taxa specialized in the colonization of a specific *Apis* species (Figure 4). As already depicted in Figure 3, the gut microbiota of *A. mellifera* and *A. cerana* showed significant differences in their microbiota composition, which were also confirmed by the intraspecies variability of *B. asteroides* (Figure 4). In this context, *B*. sp. nov. CL2 represented 77% of bifidobacterial DNA in *A. cerana*, highlighting the predominance of this single CL, while in *A. mellifera*, no data regarding CL2 was identified. Nevertheless, the distribution of *B. asteroides*‐related taxa in *A. mellifera*, with the exception of CL2, followed the data reported above, with most abundant CLs represented by *B. choladohabitans* (CL3) (34%), *B. polysaccharolyticum* (CL8) (19%), and *B. apousia* (CL5) (17%). Furthermore, *B. mizhiense* (CL4) was the only *B. asteroides*‐related taxa identified with the same abundance of 7% in both species of *Apis*, while *B*. sp. nov. CL7 was the rarest taxon with an abundance of 1% in *A. mellifera*.

**FIGURE 5:**
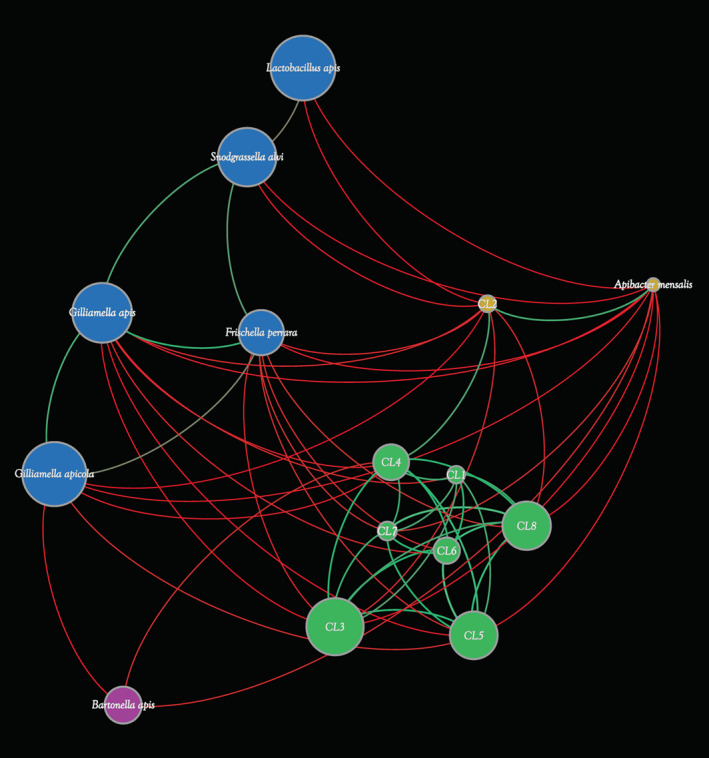
Network analysis based on the correlation of identified main microbial constituent of the honey bee gut microbiota. Circles represent the main taxa, of which *Bifidobacterium asteroides*‐related taxa are reported as CLs (CL1–CL8), and their diameter is proportional to their average abundance in the honey bee gut microbiomes. Each colour indicates a different cluster generated through positive interactions (green lines) and negative interactions (red lines).

A network based on their abundances was produced to unravel associations between the above‐described taxa (Figure 5), using data of negative and positive significant correlations retrieved from the taxonomic classification of *Apis* microbiomes. The analysis highlighted positive correlations between the most prevalent taxa represented by *Frischella perrara*, *Gilliamella apicola*, *Gilliamella apis*, *Lactobacillus apis* and *Snodgrassella alvi*, verifying an interconnection among the core microbiota of honey bee (Figures 3 and 5). Furthermore, positive correlations revealed complex interconnections between all *B. asteroides*‐related CLs except for CL2, constituting the core *Bifidobacterium* taxa of *A. mellifera* (Figure 5). Instead, CL2 displayed positive correlations only with CL4 and *Apibacter mensalis*, representing those taxa inhabiting the hindgut of *A. cerana*. Thus, the correlation analysis revealed a pattern of multi‐bifidobacterial taxa within the gut of *A. mellifera* that co‐exist with the host, while in *A. cerana*, a simple distribution characterized by members of CL2 (Figure 5). To understand the completely different pattern of bifidobacteria inhabiting the gut of the two species of *Apis*, further investigation on their genomic characteristics may unveil their specificity with the host.

### Reconstruction of *B. asteroides*‐related species and their degradative capability

The shotgun metagenomic data of honey bee gut was submitted to genome reconstruction, allowing the collection of 990,385,248 bases of bifidobacterial DNA. The reconstruction of *B. asteroides*‐related chromosomal sequences revealed 391 partially reconstructed genomes whose size was >500 Kb, of which 151 belonged to members of CL3 (Table S5). On the contrary, five and seven partially reconstructed genomes were classified as members of CL7 and CL6, followed by 17 genomes of CL1, reflecting their lower average abundance and prevalence identified between samples (Table S5).

Partially reconstructed *B. asteroides*‐related genomes with completeness >80% and contamination <3% (*n* = 63) were then employed to validate the eight CLs defined through genomic analyses. A further pangenome analysis was performed based on *B. asteroides*‐related coding sequences allowing the generation of a phylogenetic tree extending the eight clusters except for CL6 and CL7 (Figure S2). More in detail, the analysis highlighted the predominance of specific CLs within the gut of *A. mellifera* and *A. cerana*, represented by *B. choladohabitans* (CL3), *B*. sp. nov. CL2, and *B. polysaccharolyticum* (CL8), which are in high abundance in certain metagenomes and thus allowed the reconstruction of nearly complete genome sequences. As reported in Figure S2, the existence of a *B*. sp. nov. CL2 was validated, including 30 genomes of high quality to the analysis, while no additional high‐quality genomes were reconstructed for CL7. Notably, since no near‐complete *B. asteroides* (CL6) genome sequences were reconstructed, the analysis uncovered again that the common ecological attribution of this species to honey bee had been an erroneous simplification of an actual more complex bifidobacterial ecosystem of *A. mellifera*.

**FIGURE 6:**
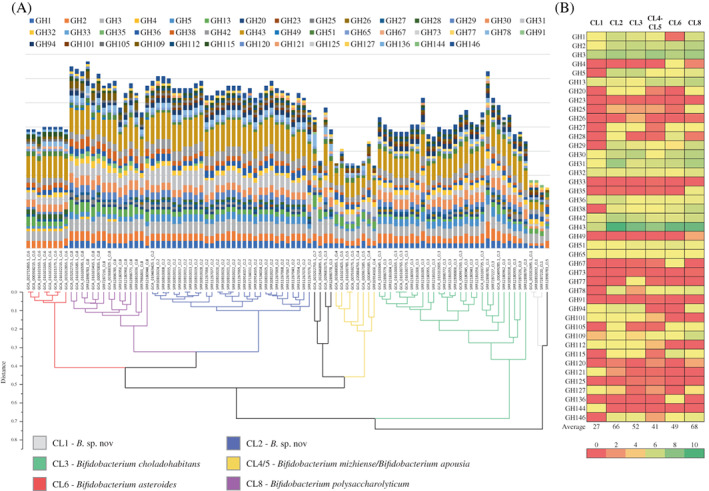
Predicted glycobiome of *Bifidobacterium asteroides*‐related taxa. (A) Depicts a hierarchical clustering based on predicted GH families of the 98 *B. asteroides*‐related strains retrieved from NCBI and reconstructed in this work through honey bee gut microbiomes. The predicted GH arsenal for every analysed bifidobacterial strain is represented by a bar plot, while *B. asteroides*‐related clusters are highlighted with different colours. (B) Displays a heat map with the average number of predicted genes encoding GHs in each CL.

Coding sequences of reclassified *B. asteroides*‐related taxa (*n* = 35) and assembled high‐quality genomes (*n* = 63) were used to access the metabolic signature of bifidobacteria inhabiting the gut of *A. mellifera* and *A. cerana* aiming to dissect their carbohydrate‐active enzyme repertoire. Identified genes encoding glycosyl hydrolases (GHs) were used to cluster together the 98 bifidobacterial strains resulting in the establishment of six enzymatic clusters (ECs) that resemble the distribution of predicted CLs (Figure 6). More in specific, members of CL1, CL2, CL3, CL6 and CL8 generated their own EC, highlighting similar GH profiles, while members of CL4 and CL5 clustered together in a common EC. Notably, the single strain representing *B*. sp. nov. CL7 was excluded from the analysis. Altogether a consistent GH core was identified between all *B. asteroides*‐related strains, encompassing GH43, GH3, GH31, GH2, GH42, GH30, GH13, GH32 and GH36 (Figure 6), reflecting metabolic activities towards a broad range of simple carbohydrates such as sucrose, glucose, fructose, galactose, ribose, mannose, melibiose, raffinose, xylose and cellobiose (Bottacini et al., 2012;Chen et al., 2021; Li et al., 2022). Recently, it has been demonstrated that an increase of sugars in flower nectar, the honey bee food, resulted in an increase in the relative abundance of bifidobacteria within the microbiome driven by their high‐grown performance on simple carbohydrates (Wang et al., 2020). Notably, *B*. sp. nov. CL1 was characterized by the lowest number of genes encoding GHs, that is, 27, and the unique presence of GH144 encoding for enzymes that hydrolyze glycosidic linkages in glucans. In contrast, *B. polysaccharolyticum* (CL8) and *B*. sp. nov. CL2 showed the highest repertoire of GHs, that is, 68 and 66, respectively (Figure 6). While members of CL8 were characterized by the unique presence of GH35 encoding galactosidase enzymes, members of CL2 were not characterized by the presence of any unique GH signature. In addition, members of *B. choladohabitans* (CL3) were characterized by the unique presence of GH77 encoding for an amylomaltase, while members of *B. asteroides* (CL6) possessed a unique repertoire of GHs, represented by GH4, GH67 and GH136 associated with genes encoding α‐glucosidase, α‐galactosidase, α‐glucuronidase and lacto‐*N*‐biosidase.

Together with GHs, the genes encoding for polysaccharide lyases (PLs) and carbohydrate esterases (CEs) repertoire were also predicted from the genomes of the 98 *B. asteroides*‐related strains revealing the absence of any specific PLs and a conserved CEs encoding gene among CLs (Table S6). In detail, a single copy of CE1 and CE9 was shared between all genomes representing a core element of *B. asteroides*‐related strains, revealing no difference between the eight CLs. Moreover, the genomes of all members of CL2 and CL6 encompassed a copy of CE8 encoding gene for a pectin methylesterase, representing a species‐specific CE. Furthermore, only a slight difference between species was identified for CL3 encoding genes, where half of the genomes of the members harboured a copy of a CE2 encoding for an acetyl xylan esterase.

The hindgut of *A. mellifera* is distinguished by the coexistence of a plethora of different bifidobacterial species, each characterized by the usage of unique GHs to complement their ability to compete in an ecological niche rich in simple sugars. In contrast, members of CL2 that specifically colonize the gut of *A. cerana* did not exhibit any unique GH signature. Instead, they shared with CL6 members the specific EC8 encoding gene for a pectin methylesterase.

## CONCLUSIONS

For years many bifidobacterial strains have been erroneously classified as belonging to the *B. asteroides* taxon, resulting in an overestimation of this species inhabiting the hindgut of honey bees through metagenomic taxonomic profiling. Here, we characterize the existence of eight species among the so‐called *B. asteroides* taxon by using genomics, metagenomics, and phylogenetics analyses. While five of them were validated by the characterization of isolates (Chen et al., 2021; Li et al., 2022), three are still missing a conventional classification. Interestingly, investigating their distribution among honey bee microbiomes, one unclassified species was found predominant in the gut of *A. cerana* and utterly absent in *A. mellifera*, opening a new insights regarding bifidobacterial species distribution among honey bees and their coevolution with the host. Notably, the host‐specificity of such *B. asteroides*‐related species may represent potential valuable novel candidates of next generation of probiotics for honey bees. Furthermore, a glycobiome analysis, encompassing reclassified *B. asteroides*‐related taxa and assembled high‐quality genomes, revealed a conserved repertoire of GHs reflecting their metabolic behaviours towards a broad range of simple carbohydrates abundant in the gut of honey bees.

## AUTHOR CONTRIBUTIONS

Gabriele Andrea Lugli performed bioinformatics analyses, wrote the manuscript and designed the study; Federico Fontana and Chiara Tarracchini managed the metadata and data results; Leonardo Mancabelli performed the statistical analyses; Christian Milani and Francesca Turroni supervised the project and edited the manuscript; Marco Ventura supervised the project and designed the study.

## CONFLICT OF INTEREST

The authors declare that they have no competing interests.

## Supporting information


**Figure S1.** Selection of unclassified bifidobacterial species related to *Bifidobacterium asteroides*. The hierarchical clustering is based on an ANI values matrix of all 187 genome sequences collected from the NCBI database. The additional 26 genomes identified as related to the *B. asteroides* taxon are highlighted in green.

**Figure S2.** Phylogenomic tree of 98 *Bifidobacterium asteroides*‐related strains based on the concatenation of core protein sequences of reconstructed genomes retrieved from the metagenomes of the hindgut of honey bees. Different colours highlight the division into eight taxa (named from CL1 to CL8). The phylogenetic tree was constructed by the neighbour‐joining method, with the genome sequence of *Bifidobacterium actinocoloniiforme* DSM 22766 as an outgroup. Bootstrap percentages above 50 are shown at node points based on 1000 replicates of the phylogenetic tree.

**Table S1.** Unclassified bifidobacteria in the NCBI repository
**Table S2.** Quality control of *Bifidobacterium asteroides*‐related strains
**Table S3.** Shotgun metagenomic samples of Apis mellifera and Apis cerana
**Table S4.** Filtering table of shotgun metagenomics data
**Table S5.** Quality control of reconstructed *Bifidobacterium asteroides*‐related strains
**Table S6.** Carbohydrate esterase (CE) profiling of *Bifidobacterium asteroides*‐related strains

## Data Availability

Raw sequences of shotgun metagenomics experiments are accessible through SRA study listed in Table S3. In addition, genome sequences used in this project have been reported in Tables 1 and S1.
