# Supplementary figures and images for: Exploring the biodiversity of Bifidobacterium asteroides among honey bee microbiomes
Source: Environ Microbiol. 2022 Oct 3;24(12):5666–79. doi: 10.1111/1462-2920.16223 (PMC10092428; doi:10.1111/1462-2920.16223)

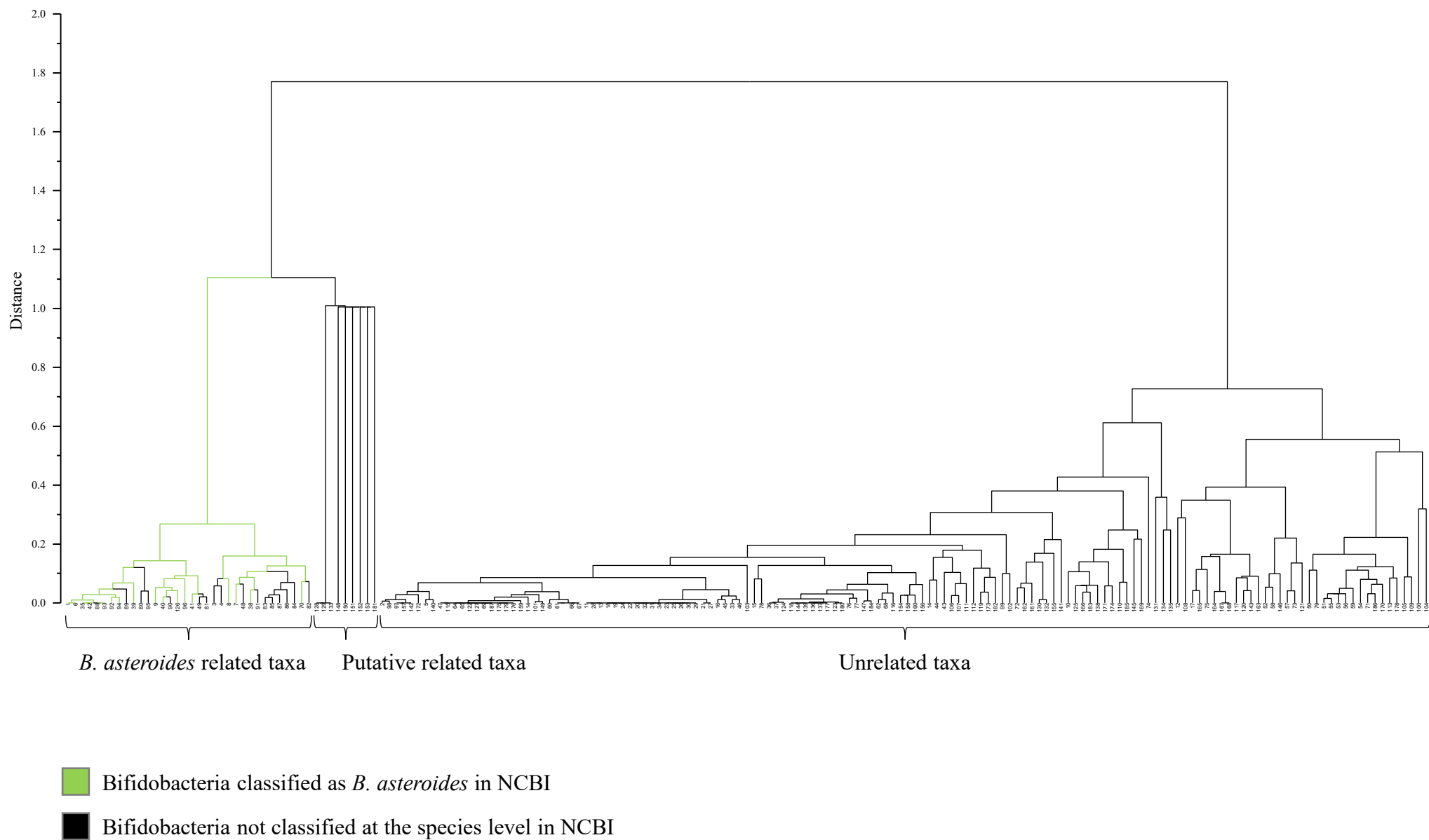

**Figure S1**

Supplement: Supplementary file 1 — Figure S1. Selection of unclassified bifidobacterial species related to Bifidobacterium asteroides. The hierarchical clustering is based on an ANI values matrix of all 187 genome sequences collected from the NCBI database. The additional 26 genomes identified as related to the B. asteroides taxon are highlighted in green. [file EMI-24-5666-s001.pdf]
