# Supplementary material for: Exploring the biodiversity of Bifidobacterium asteroides among honey bee microbiomes
Source: Environ Microbiol. 2022 Oct 3;24(12):5666–79. doi: 10.1111/1462-2920.16223 (PMC10092428; doi:10.1111/1462-2920.16223)
Supplement: Supplementary file 2 — Figure S2. Phylogenomic tree of 98 Bifidobacterium asteroides‐related strains based on the concatenation of core protein sequences of reconstructed genomes retrieved from the metagenomes of the hindgut of honey bees. Different colours highlight the division into eight taxa (named from CL1 to CL8). The phylogenetic tree was constructed by the neighbour‐joining method, with the genome sequence of Bifidobacterium actinocoloniiforme DSM 22766 as an outgroup. Bootstrap percentages above 50 are shown at node points based on 1000 replicates of the phylogenetic tree. [file EMI-24-5666-s003.pdf]

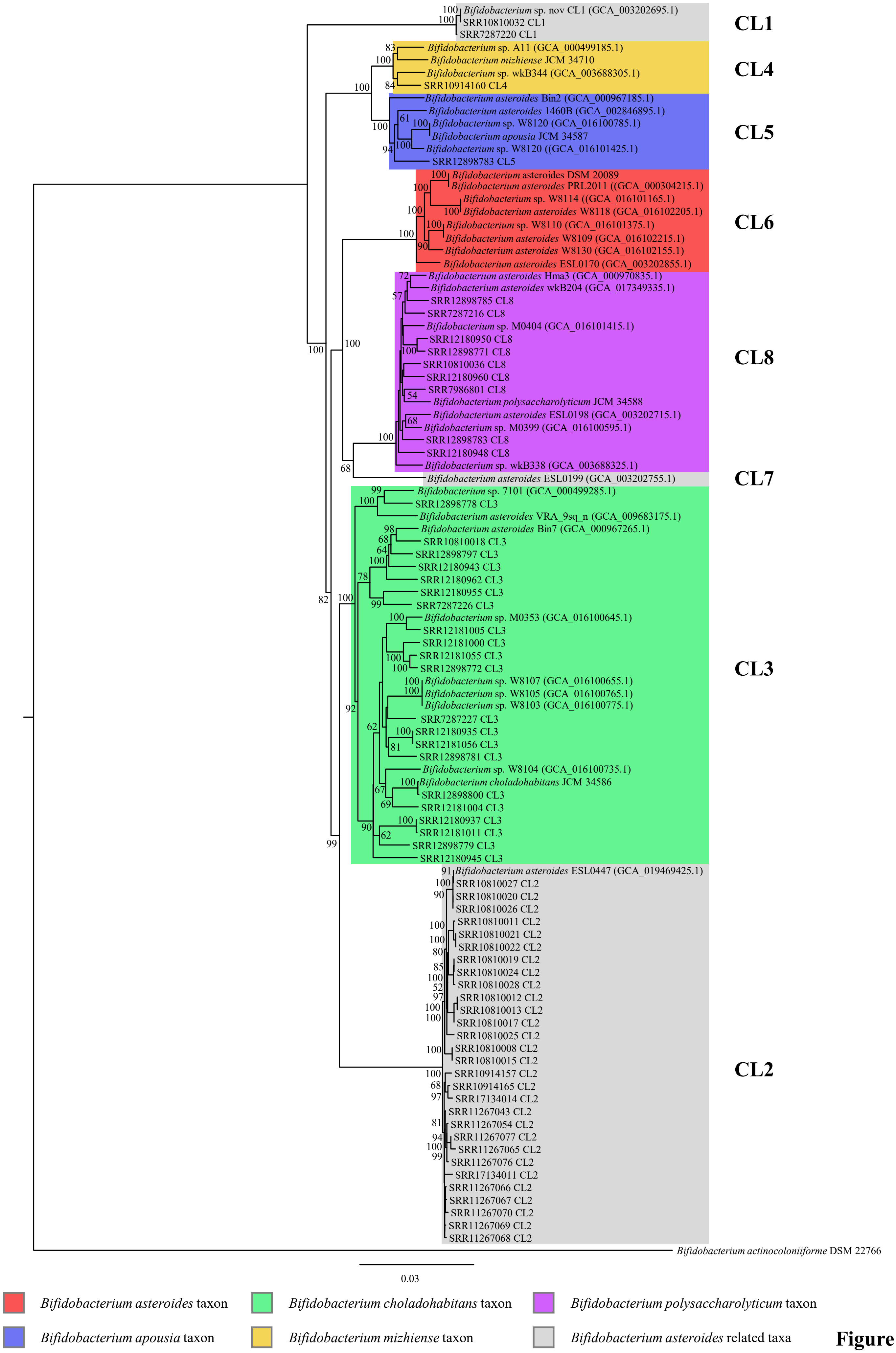
Bifidobacterium sp. nov CL1 (GCA_003202695.1)
SRR10810032 CL1
SRR7287220 CL1
CL1
Bifidobacterium sp. A11 (GCA_000499185.1)
Bifidobacterium mizhiense JCM 34710
Bifidobacterium sp. wkB344 (GCA_003688305.1)
SRR10914160 CL4
CL4
Bifidobacterium asteroides Bin2 (GCA_000967185.1)
Bifidobacterium asteroides 1460B (GCA_002846895.1)
Bifidobacterium sp. W8120 (GCA_016100785.1)
Bifidobacterium apousia JCM 34587
Bifidobacterium sp. W8120 ((GCA_016101425.1)
SRR12898783 CL5
CL5
Bifidobacterium asteroides DSM 20089
Bifidobacterium asteroides PRL2011 ((GCA_000304215.1)
Bifidobacterium sp. W8114 ((GCA_016101165.1)
Bifidobacterium asteroides W8118 (GCA_016102205.1)
Bifidobacterium sp. W8110 (GCA_016101375.1)
Bifidobacterium asteroides W8109 (GCA_016102215.1)
Bifidobacterium asteroides W8130 (GCA_016102155.1)
Bifidobacterium asteroides ESL0170 (GCA_003202855.1)
CL6
Bifidobacterium asteroides Hma3 (GCA_000970835.1)
Bifidobacterium asteroides wkB204 (GCA_017349335.1)
SRR12898785 CL8
SRR7287216 CL8
Bifidobacterium sp. M0404 (GCA_016101415.1)
SRR12180950 CL8
SRR12898771 CL8
SRR10810036 CL8
SRR12180960 CL8
SRR7986801 CL8
Bifidobacterium polysaccharolyticum JCM 34588
Bifidobacterium asteroides ESL0198 (GCA_003202715.1)
Bifidobacterium sp. M0399 (GCA_016100595.1)
SRR12898783 CL8
SRR12180948 CL8
Bifidobacterium sp. wkB338 (GCA_003688325.1)
CL8
Bifidobacterium asteroides ESL0199 (GCA_003202755.1)
CL7
Bifidobacterium sp. 7101 (GCA_000499285.1)
SRR12898778 CL3
Bifidobacterium asteroides VRA_9sq_n (GCA_009683175.1)
Bifidobacterium asteroides Bin7 (GCA_000967265.1)
SRR10810018 CL3
SRR12898797 CL3
SRR12180943 CL3
SRR12180962 CL3
SRR12180955 CL3
SRR7287226 CL3
Bifidobacterium sp. M0353 (GCA_016100645.1)
SRR12181005 CL3
SRR12181000 CL3
SRR12181055 CL3
SRR12898772 CL3
Bifidobacterium sp. W8107 (GCA_016100655.1)
Bifidobacterium sp. W8105 (GCA_016100765.1)
Bifidobacterium sp. W8103 (GCA_016100775.1)
SRR7287227 CL3
SRR12180935 CL3
SRR12181056 CL3
SRR12898781 CL3
Bifidobacterium sp. W8104 (GCA_016100735.1)
Bifidobacterium choladohabitans JCM 34586
SRR12898800 CL3
SRR12181004 CL3
SRR12180937 CL3
SRR12181011 CL3
SRR12898779 CL3
SRR12180945 CL3
CL3
Bifidobacterium asteroides ESL0447 (GCA_019469425.1)
SRR10810027 CL2
SRR10810020 CL2
SRR10810026 CL2
SRR10810011 CL2
SRR10810021 CL2
SRR10810022 CL2
SRR10810019 CL2
SRR10810024 CL2
SRR10810028 CL2
SRR10810012 CL2
SRR10810013 CL2
SRR10810017 CL2
SRR10810025 CL2
SRR10810008 CL2
SRR10810015 CL2
SRR10914157 CL2
SRR10914165 CL2
SRR17134014 CL2
SRR11267043 CL2
SRR11267054 CL2
SRR11267077 CL2
SRR11267065 CL2
SRR11267076 CL2
SRR17134011 CL2
SRR11267066 CL2
SRR11267067 CL2
SRR11267070 CL2
SRR11267069 CL2
SRR11267068 CL2
CL2
Bifidobacterium actinocoloniiforme DSM 22766
0.03
Bifidobacterium asteroides taxon
Bifidobacterium choladohabitans taxon
Bifidobacterium polysaccharolyticum taxon
Bifidobacterium apousia taxon
Bifidobacterium mizhiense taxon
Bifidobacterium asteroides related taxa

**Figure S2**
